# Surgical Management of Type II Odontoid Fractures in a Resource-Limited Setting: A Case Series

**DOI:** 10.7759/cureus.66585

**Published:** 2024-08-10

**Authors:** Ntsambi Glennie, Israël A Maoneo, Kisubi Michel, Chérubin Tshiunza, Antoine Beltchika

**Affiliations:** 1 Neurosurgery, University of Kinshasa, Kinshasa, COD; 2 Neurosurgery, University of Kisangani, Kisangani, COD; 3 Neurosurgery, Centre Hospitalier Initiative Plus, Kinshasa, COD

**Keywords:** anderson and d'alonzo classification, atlas posterior arch resection, odontoid screw, anterior odontoid fixation, odontoid fracture

## Abstract

Type II odontoid fracture, classified by Anderson and D’Alonzo, is the most common traumatic injury to the odontoid process. Surgical management of this lesion is particularly challenging in underresourced countries. This study aims to report the preliminary experience of the Kinshasa University Teaching Hospital in Kinshasa, Democratic Republic of the Congo, particularly using adaptive techniques. Three patients, aged 22, 30, and 32 years, respectively, were admitted to the neurosurgery department with Anderson and D'Alonzo type II odontoid fractures as confirmed by CT scan imaging. The first two patients underwent anterior odontoid fixation using a non-cannulated orthopaedic screw with an image intensifier. In the third case, partial resection of the C1 posterior arch was performed, followed by immobilisation using a rigid Philadelphia neck brace. Postoperative follow-up in all three cases was uneventful, and neurological outcomes were satisfactory. Odontoid surgery remains challenging for developing countries. The use of a non-cannulated orthopaedic screw for anterior fixation and posterior spinal cord decompression via partial resection of the C1 posterior arch, followed by external cervical immobilisation with a rigid neck brace for neglected fractures, could be effective alternatives to conventional surgical techniques. However, randomised multicentre studies are required to confirm the efficacy and safety of these techniques.

## Introduction

Odontoid fracture is a common fracture of the cervical spine, occurring in 10 to 20% of cases [1]. Anderson and D'Alonzo type II fracture is the most common (65%-74%) and the most mechanically unstable [2]. Surgical treatment is often necessary, as conservative treatment is frequently complicated by non-union due to poor vascularization and several mechanical constraints that render the fracture unstable [3,4]. This instability can lead to malunion, resulting in late cervical myelopathy [4]. Surgical management involves either anterior or posterior fixation after odontoid fracture reduction, facilitated by sophisticated devices such as neuro-navigation and O-arm [5,6].

In the anterior approach, the odontoid is reduced and fixed with one or two screws. This technique, initially practised by the Japanese surgeon Nakanishi in 1980 [7] and subsequently developed by Böhler in 1982 [8], involves approaching the cervical spine through the classic anterior approach, reducing the fracture, and maintaining the reduction by placing the screw. We typically use Lag, Herbert, and Acutrak universal cannulated odontoid screws [9]. The odontoid screw fixation technique is not recommended for children under six years old but is indicated for children aged six to 18 years [10]. A transarticular C1/C2 fixation can also be performed via the anterior approach [11].

The type of inferior posterior fracture and concomitant transverse ligament tear are selected for posterior fixation. Historically, Brooks and Gallie performed posterior cervical fusion using sublamellar wires, and later, Dickman and Sonntag's methods incorporated the use of sublaminar wires and bone grafts, achieving an 86% fusion rate. This was followed by a transarticular screw technique described by Jeanneret and Magerl, providing almost 100% fusion. The Goel-Harms technique is an excellent alternative to anterior fixation, involving the placement of polyaxial screws in the lateral masses of C1 and pedicle screws at the level of C2, and is a valuable alternative to the Magerl technique [12].

In cases of ankylosing spondylitis, diffuse idiopathic skeletal hyperostosis (DISH), or complex lesions, C1/2 stabilisation alone is insufficient, and posterior occipito-cervical stabilisation and fusion (C0-C3/4) are required [11]. The algorithm for this surgical management was summarised by Carvallo and colleagues [12].

Despite advanced equipment, there is still controversy in well-equipped neurosurgical centres between proponents of anterior odontoid screwing and advocates of the posterior approach [13, 14]. This study aims to report the preliminary experience of the neurosurgery department of Kinshasa University Teaching Hospital, Kinshasa, Democratic Republic of the Congo, regarding the anterior fixation of two cases of type II odontoid fracture using non-cannulated orthopaedic screws and the partial resection of the posterior arch of the atlas, followed by rigid external immobilisation for late myelopathy in a neglected type II odontoid fracture.

## Case presentation

Case one

**Figure 1:**
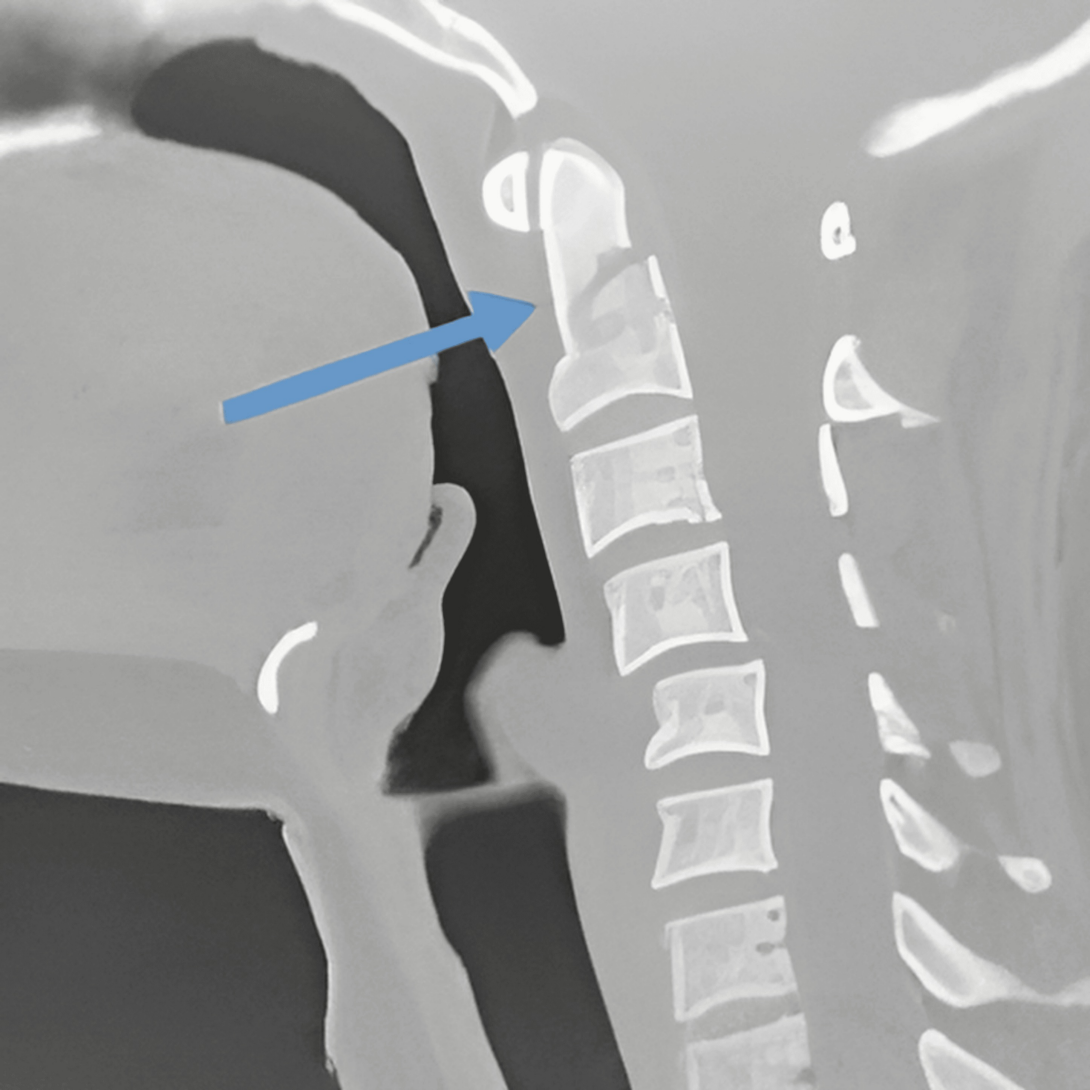
The CT scan shows a type II odontoid fracture in the first case (blue arrow).

The patient, a 22-year-old female, was involved in a road traffic accident. A large vehicle carrying rubble with brake failure collided with her while crossing the road. She presented with neck pain, monoparesis of the right upper limb with an overall motor strength estimated at 3/5, and facial abrasions. A CT scan revealed an oblique fracture at the base of the odontoid extending to the body of the axis, along with a benign sprain at C4-C5 (Figure 1). Upon admission, two hours post-accident, she was administered corticosteroids and analgesics and fitted with a rigid, removable neck brace. Osteosynthesis via anterior fixation of the odontoid with a non-cannulated orthopaedic screw was performed two weeks post-trauma. The postoperative period was uneventful, both surgically and neurologically. She was reviewed twice in follow-up consultations within a month after discharge, and her long-term recovery remained satisfactory.

Case two

**Figure 2:**
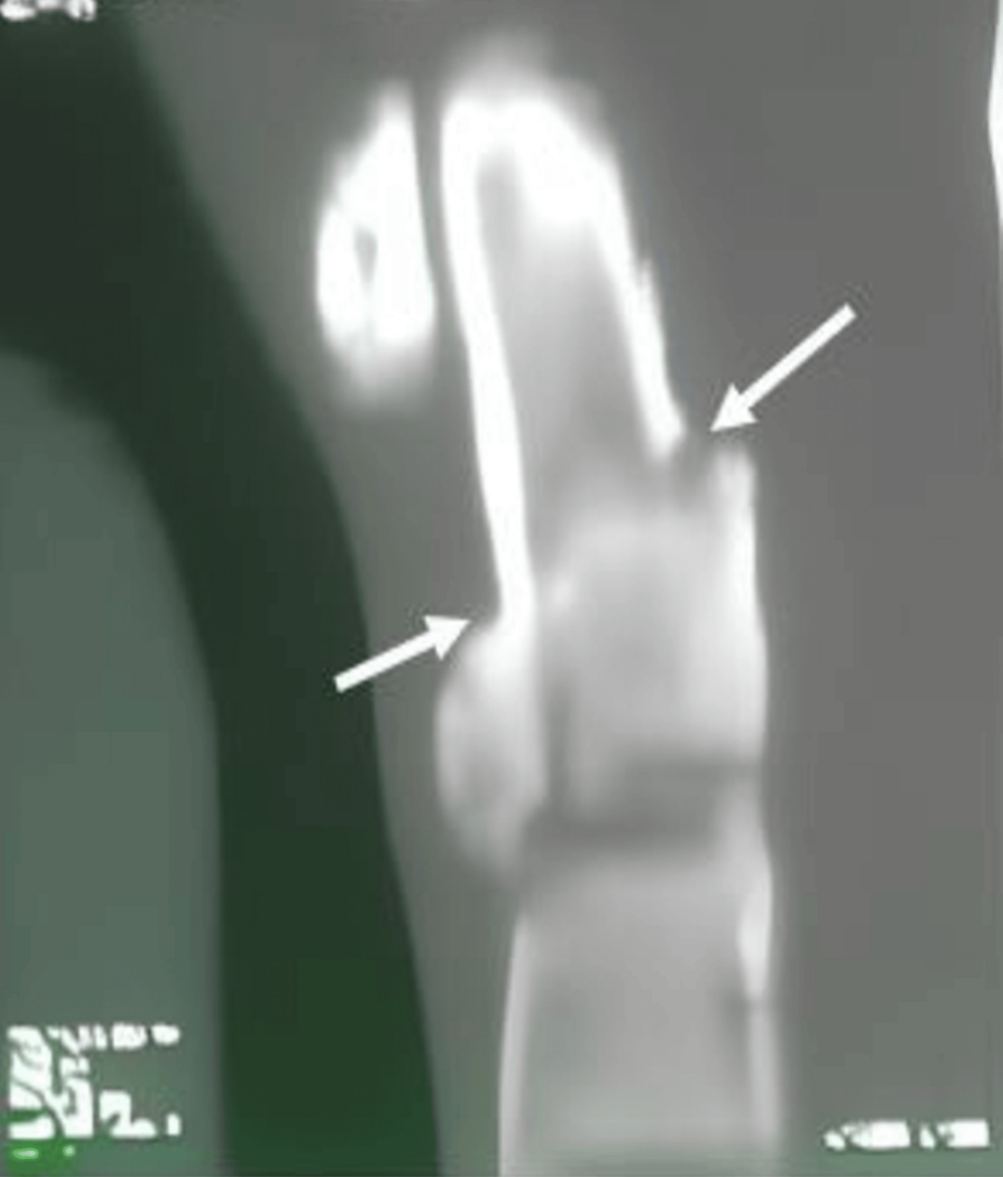
A CT shows a type II odontoid fracture in the second case (white arrows).

The patient, a 30-year-old male, was treated in our department for neck pain following an impact by a military vehicle while on guard duty at the entrance of a military camp. He experienced severe neck pain without neurological deficits. A cervical neck brace was applied and maintained until imaging and surgical treatment were performed. A CT revealed a type II odontoid fracture with a fracture of the right pedicle (Figure 2). The MRI showed no signs of intramedullary injury. Odontoid screwing using a solid orthopaedic screw was carried out one month post the accident. The neck was subsequently immobilised in a rigid Philadelphia-type neck brace. The postoperative follow-up was straightforward, both surgically and neurologically.

Surgical procedure for the first two cases

**Figure 3:**
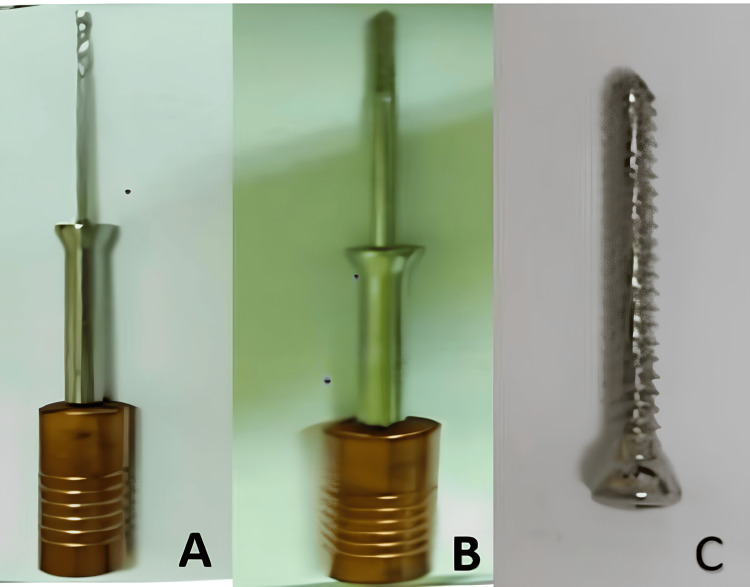
The instruments used in the surgical procedure A: tunnelizer; B: tap; C: solid screw

**Figure 4:**
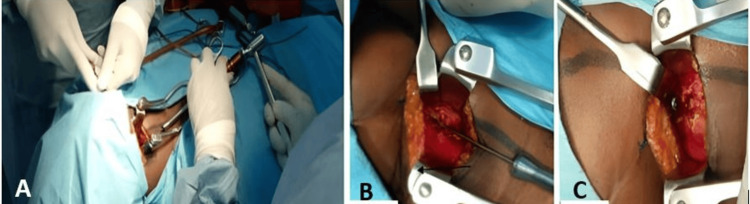
Insertion of the tunnelling device (A and B) and installation of the screw (C)

**Figure 5:**
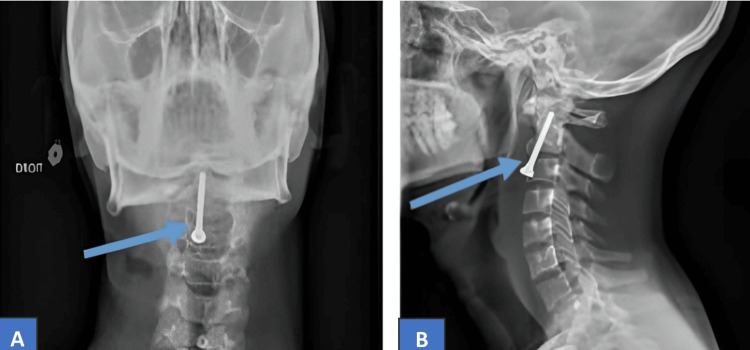
Postoperative X-rays in frontal (A) and profile (B) views show the odontoid screw locations.

The patients were positioned supine with two logs placed under their shoulders, allowing for clearance of the anterior cervical region and extension of the head, which was fixed to Mayfield's headrest after achieving alignment under fluoroscopy to facilitate screwing. A transverse incision was made, followed by dissection of the subcutaneous tissues and muscles and passage into the carotid gutter. Fracture reduction was performed under profile fluoroscopy. The C2-C3 disc was resected, and a gutter was created on the upper half of the anterior face of the C3 body. The body of the C2 and the odontoid process were drilled using a tunneller, followed by tapping with a 3 mm tap (Figure 3). Screwing was then performed under fluoroscopy control using a solid orthopaedic screw, 35 mm long and 3.5 mm in diameter (Figure 4). The wound was sutured after placing a suction drain. At the end of the procedure, the cervical spine was immobilised with a rigid Philadelphia Minerva brace. The procedure lasted approximately two hours on average. Postoperative monitoring included observing vital signs and neurological status, checking for early signs of thromboembolic diseases, monitoring for the onset of dysphagia and phonatory difficulties, and conducting an X-ray to ensure the screw was correctly positioned (Figure 5).

Case three

The patient, a 32-year-old, was admitted to the neurosurgery department for neck pain and functional impairment of all four limbs following a road accident that occurred two years prior. The patient had been hit by a speeding bus, resulting in neck pain, abrasions to the lower limbs, and an incomplete traumatic amputation of the right leg. Initially hospitalised in a peripheral centre, he received analgesics, and antibiotics, and underwent surgery to complete the supracondylar amputation of the right limb. Persistent neck pain and limb weakness following physiotherapy led to his consultation at the Cliniques Universitaires de Kinshasa (CUK) two years later.

**Figure 6:**
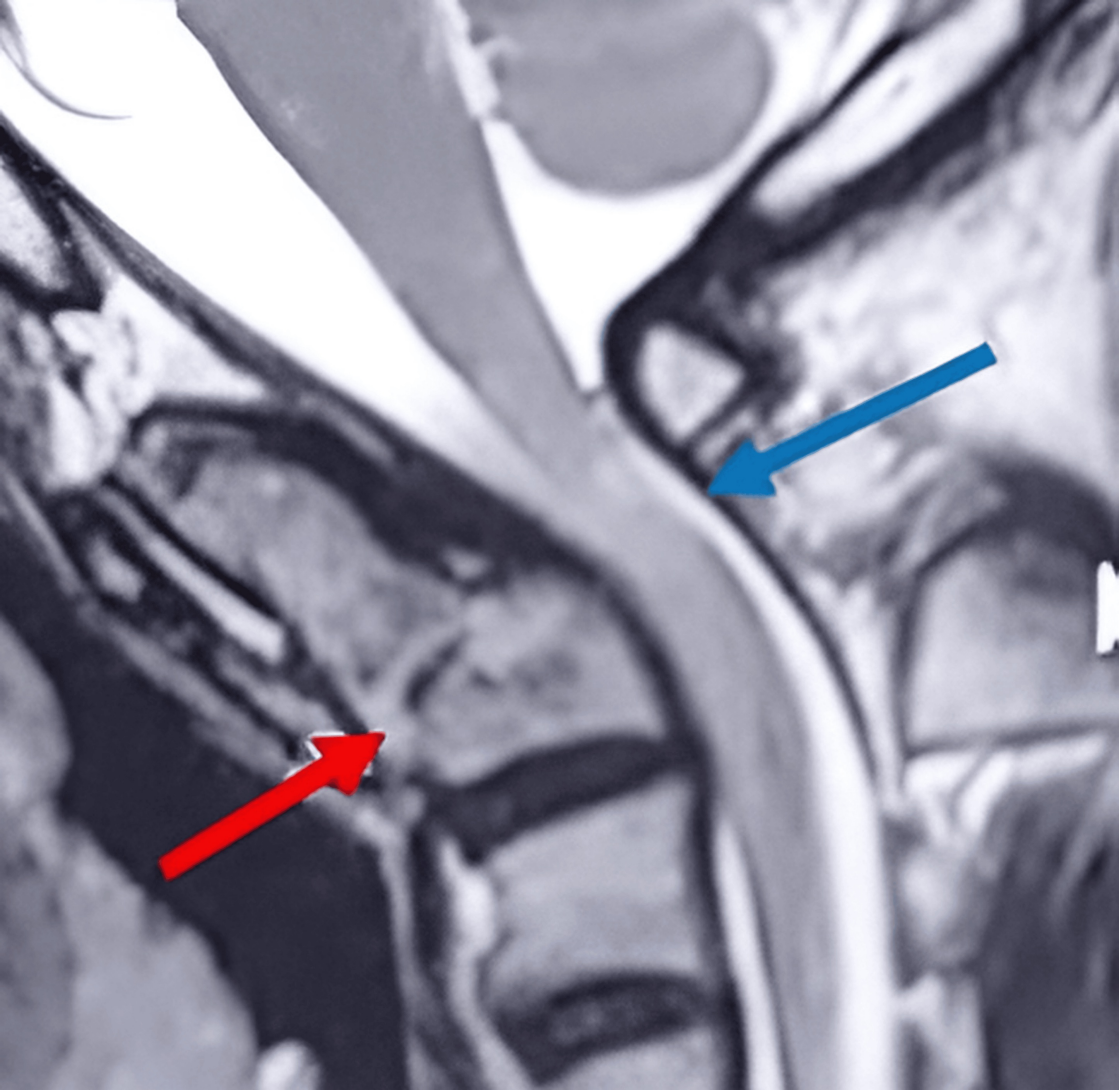
The MRI of the third case shows a type II odontoid fracture, consolidated in a vicious position (red arrow) with cervical kyphosis and complicated by myelopathy (blue arrow).

On physical examination, he exhibited a stiff neck, nuchal rigidity, and pain on posterior cervical palpation, alongside a healed right supracondylar amputation stump. Neurologically, he was lucid with isochoric and reactive pupils. The motor American Spinal Injury Association (ASIA) score was assessed at 39/100, considering the amputation of the right lower limb. There were no sphincter disorders; sensitivity was intact, and the bulbocavernosus reflex was present. Cervical MRI revealed a neglected Anderson and D'Alonzo type II fracture of the odontoid process, with a malunion showing anterior translation >2 mm and posterior angulation of 40°. The MRI also indicated an intramedullary hypersignal in the T2 sequence, consistent with cervical myelopathy due to compression at the fracture site (Figure 6).

Surgical decompression of the spinal cord was performed through partial resection of the C1 posterior arch. The postoperative period was uncomplicated. The neck was immobilised with a rigid Philadelphia brace for eight weeks. Physiotherapy commenced on the seventh postoperative day. A postoperative CT scan confirmed complete spinal cord decompression following resection of the posterior arch of the atlas. At discharge on the 15^th^ postoperative day, the ASIA score had improved from 39 to 61/100 (considering the supracondylar amputation of the right lower limb). Two months postoperatively, the patient had regained full motor function, including that of the remaining segment of the amputated limb.

Surgical procedure for the third case

**Figure 7:**
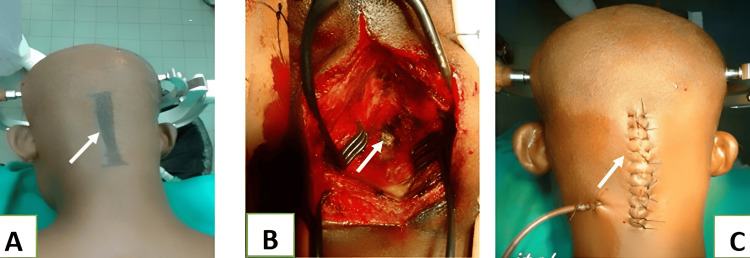
Intraoperative images A: incision line; B: surgical opening and exposure of the posterior arch of the atlas; C: wound closure (white arrows)

The patient, under general anaesthesia with oral-tracheal intubation, was positioned prone with the head stabilised using a Mayfield headrest. The operative site was cleaned with 4% betadine red scrub. The incision line was traced (Figure 7A), and the surgical site was disinfected with 10% betadine yellow before covering the patient with sterile drapes. An occipito-cervical incision was made, involving the skin and subcutaneous tissue. A progressive midline dissection was performed until the C2 spinous process, C1 tubercle, and occipital bony margin were visualised (Figure 7B). Bipolar cauterisation was utilised for haemostasis control. The dural sac was released from adhesion to the posterior arch of C1.

**Figure 8:**
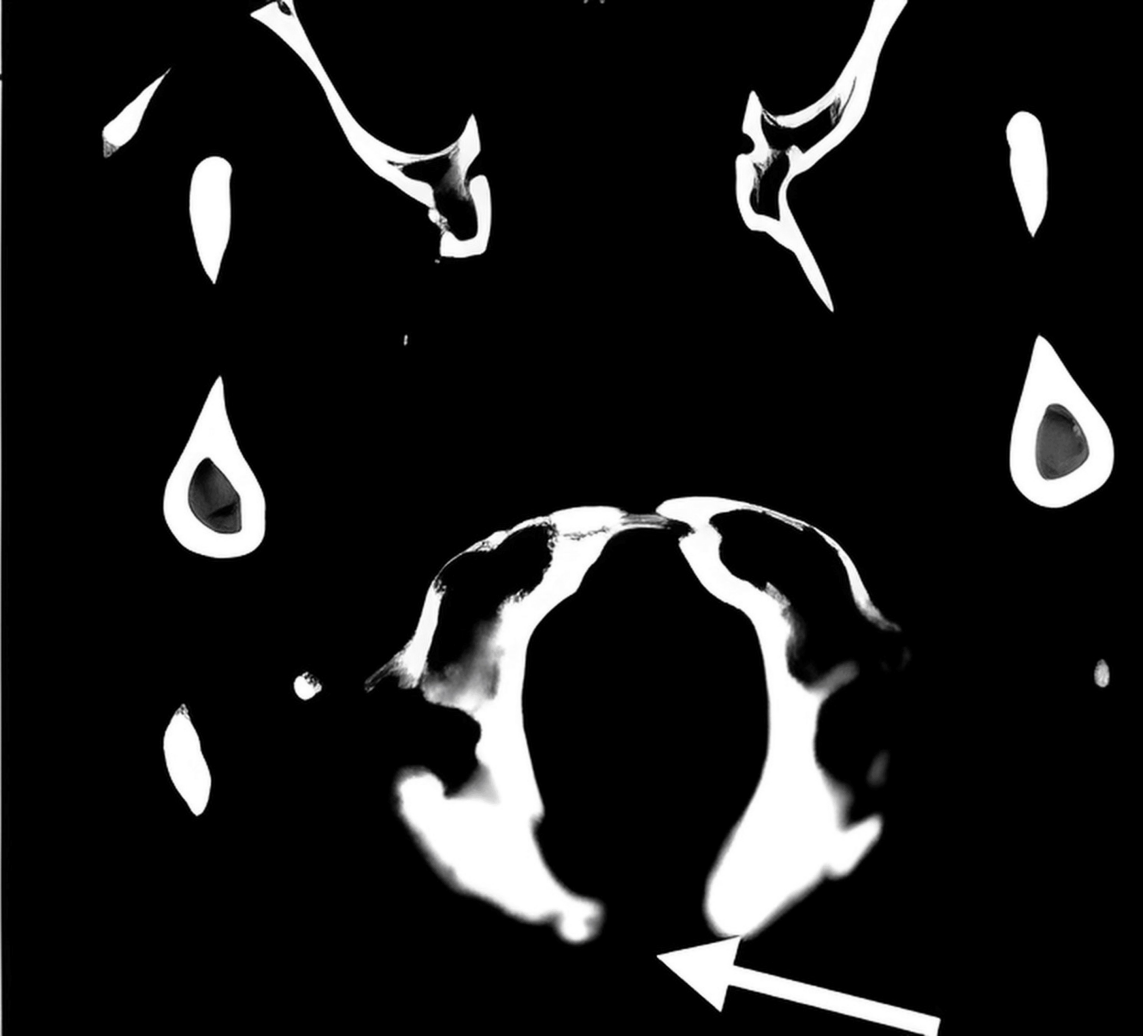
Postoperative CT images show the partial resection of the posterior arch of the atlas (white arrow).

The posterior arch of C1 was resected using fine-gouge forceps and a Kerrison rodent, maintaining a maximum distance of 1 cm on either side of the midline. The dural sac was observed to relax. Surgicel was inserted for haemostasis, and the incision was closed in layers after drain installation (Figure 7C), followed by the application of a dry, sterile dressing. The cervical spine was immobilised with a rigid Philadelphia Minerva brace. A postoperative CT scan was performed to show the extent of the resection of the posterior arch, ensuring the spinal cord was free of posterior pressure (Figure 8).

The three cases described above are summarized in Table 1.

**Table 1:** Summary of the cases

Case number	Age (years)	Sex	Trauma context	Types of lesions	Interventions	Follow-up (months)	Recovery
1	22	Female	Road traffic accident (RTA)	Type II odontoid fracture	Anterior screwing of the odontoid	48	Complete
2	30	Male	RTA	Type II odontoid fracture, fracture of the anterior arches of ribs 4, 5, and 6, fracture of the distal end of the radius	Anterior screwing of the odontoid	8	Complete
3	32	Male	RTA	Type II odontoid fracture	Resection of C1 posterior arch	36	Complete

## Discussion

This preliminary case report is the first to document odontoid surgery in our context. Additionally, to our knowledge, this is the first report in the cervical spine surgery literature to describe the use of a solid orthopaedic screw for anterior fixation and external stabilisation for neglected fractures.

Odontoid fractures are the most common type of cervical spine fractures, typically resulting from high-energy trauma such as road traffic accidents, especially in young individuals [10,13,15]. As a result, Anderson and D'Alonzo type II and III fractures, which are unstable and necessitate surgical intervention, are predominant. Stable type II or III fractures are rare, and type I fractures require conservative treatment. Fractures with proper alignment, no dynamic instability, and no neurological deficits also necessitate conservative management [10]. Most studies have reported outcomes of surgical versus conservative management in elderly patients with odontoid fractures related to osteoporosis. Few have focused on traumatic fractures, and even fewer on resource-limited settings [16].

In less equipped health facilities, such as those in the Democratic Republic of Congo, surgical fixation of odontoid fractures faces significant challenges due to the lack of specific neurosurgical implants, neuronavigation, and O-arm. Lag, Herbert, and Acutrack universal cannulated odontoid screws are unavailable [9,10]. Consequently, neurosurgeons often have to resort to conservative treatment or adaptive materials.

Anterior odontoid screw fixation, introduced by Boehler in 1982 [8], offers several advantages over posterior fixation. It restores the anatomy of the odontoid, preserves most cervical rotation, provides immediate stability to the spine, and has a higher fusion rate. It allows for an earlier return to normal activities, reduces the duration of the procedure, and carries less risk of spinal artery injury, blood loss, postoperative narcotic use, tissue damage, and hospitalisation [15]. The decision for surgical treatment is based on certain factors: integrity of the transverse ligament, minimal fracture displacement (no more than 6 mm), and good bone quality (not osteoporotic) [15]. In our series, all cases involved severe trauma due to road traffic accidents, with type II fractures, minimal displacement, and a mean patient age of 23.6 years. In the first two cases, we used solid, fully threaded orthopaedic screws with a small short head, 3.5 cm long and 3.5 mm in diameter, to overcome the limitations of the under-equipped operating theatre. Postoperative check-ups showed satisfactory radiological results.

Complications are rare with anterior fixation, but dysphagia and pneumonia can occur postoperatively [15]. Increased X-ray exposure due to the use of fluoroscopy is also a concern [17]. In all three cases, we did not observe any complications among our operated patients.

In the third case, we partially resected the posterior arch of the atlas, followed by immobilisation using a rigid neck brace. This resection technique is delicate due to the surgical area's anatomy. The posterior arch of the atlas supports the vertebral arteries along its upper edge before they enter the cranium. The surgeon should resect only the middle part and avoid extending laterally, not exceeding 1 cm on each side. Previous studies by Jeon et al. [18], Shabaan et al. [19], and Shamji et al. [20] performed posterior spinal cord decompression by partially resecting the posterior arch of the atlas but used internal immobilisation with screws and plates.

The principle of surgical management for the late neurological deficit in myelopathy following a neglected odontoid fracture involves decompression and fixation. However, there is still debate over the best approach. Should decompression and stabilisation be performed entirely anteriorly [1], entirely posteriorly [18], or using a combination of both? The choice of surgical technique has traditionally been left to the surgeon's discretion.

The anterior approach involves transoral decompression by resecting or reducing the odontoid process, followed by anterior fixation. The posterior approach involves partial resection of the C1 posterior arch followed by posterior, occipito-cervical, or cervical fixation [12]. The third technique combines the anterior and posterior approaches, with the surgeon performing either anterior decompression alone or combined with posterior decompression followed by posterior occipito-cervical fixation [12, 19]. In our patient's case, resection was not followed by internal fixation due to a lack of adequate equipment. Instead, we used external immobilisation with a rigid Philadelphia-type neck brace. The better clinical outcome observed in this case without internal posterior fixation could be attributed to two factors: the odontoid fracture was already healed despite its malalignment, and the displacement of the consolidated fracture elements (the atlas, the dens, and the body of the axis) was minimal.

## Conclusions

Odontoid surgery remains a challenge for under-equipped countries, necessitating the use of adaptive strategies to overcome surgical obstacles. The use of a solid orthopaedic screw for anterior fixation and posterior spinal cord decompression by partial resection of the posterior arch of C1 followed by rigid external cervical immobilisation for a neglected fracture can be effective alternatives to conventional surgical techniques. However, randomised, multicentre studies are needed to confirm the efficacy and safety of these techniques.
